# Downregulation of BIRC2 hinders the progression of rheumatoid arthritis through regulating TRADD

**DOI:** 10.1002/iid3.978

**Published:** 2023-10-04

**Authors:** Yanting Rao, Shengjing Xu, Ting Lu, Yuanyuan Wang, Manman Liu, Wei Zhang

**Affiliations:** ^1^ Department of Rheumatology and Immunology The Affiliated Jiangning Hospital of Nanjing Medical University Nanjing China

**Keywords:** BIRC2, necroptosis, rheumatoid arthritis, TRADD

## Abstract

**Aim:**

Rheumatoid arthritis (RA) is a chronic inflammation mediated by an autoimmune response. Baculoviral IAP repeat‐containing 2 (BIRC2) and tumor necrosis factor receptor 1‐associated death domain protein (TRADD) have been reported to be highly expressed in RA, while their specific roles during RA progression remain unclear. This study aims to explore the specific regulation of BIRC2/TRADD during the progression of RA.

**Methods:**

C28/I2 cells were stimulated by lipopolysaccharide (LPS) to establish an in vitro RA cellular model. The expression level of BIRC2 and TRADD was examined by quantitative real‐time polymerase chain reaction and western blot. Cell Counting Kit‐8 and flow cytometry assays were performed to examine cell viability and necroptosis, respectively. The oxidative stress markers were detected using commercial kits, and the pro‐inflammatory cytokines were measured by ELISA assay. The interaction between BIRC2 and TRADD was verified by co‐immunoprecipitation assay.

**Results:**

BIRC2 and TRADD were discovered to be highly expressed in LPS‐mediated C28/I2 cells. BIRC2 knockdown was demonstrated to inhibit LPS‐induced cell viability loss, necroptosis, oxidative stress, and inflammation in C28/I2 cells. BIRC2 could interact with TRADD and positively regulate TRADD expression. In addition, the protective role of BIRC2 knockdown against LPS‐mediated injuries in C28/I2 cells was partly weakened by TRADD overexpression.

**Conclusion:**

In summary, BIRC2 knockdown alleviated necroptosis, oxidative stress, and inflammation in LPS‐mediated C28/I2 cells, which might correlate to the regulatory role of TRADD, indicating a novel target for the treatment of RA.

## Graphical abstract

Baculoviral IAP repeat‐containing 2 (BIRC2) knockdown could attenuate the progression of rheumatoid arthritis by inhibiting inflammation, oxidative stress, and necroptosis. BIRC2 knockdown might play its protective role against lipopolysaccharide‐mediated C28/I2 cells through downregulating tumor necrosis factor receptor 1‐associated death domain protein.

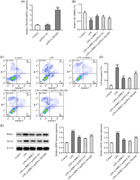

## 1 INTRODUCTION

Rheumatoid arthritis (RA) is a common systemic inflammatory autoimmune disease manifested as synovial inflammation, pannus formation, progressive bone erosion, and joint destruction.^1^ In addition to joint damage, RA also harms extra‐articular organs, such as the skin, lung, heart, and nervous system.^2^ RA is more prevalent at the age of 50–60 years and females are more susceptible than men.^3^ To date, the key therapeutic approaches to RA are surgeries and drugs, including disease‐modifying antirheumatic drugs (DMARDs) and nonsteroidal anti‐inflammatory drugs (NSAIDs). Accumulating evidence reveals that massive permanent joint damage occurs within the first 2 years, if untreated within 3–6 months after the onset of RA.^4^, ^5^ Thus, it is essential to develop reliable biomarkers and effective treatments for RA.

Currently, the exact etiology of RA has not been fully understood yet, and genetic, environmental, microbial, metabolic, and immune factors have been confirmed to jointly contribute to the initiation and development of RA.^6^, ^7^, ^8^ As a prototype of autoimmune diseases, oxidative stress has been recognized as an important pathophysiology of RA.^9^ Of note, the involvement of necroptosis during RA progression attracts much attention in recent years and necroptosis may act as promising target for innovative therapy in RA.^10^, ^11^

The cellular inhibitor of apoptosis protein 1 (cIAP1), also known as baculoviral IAP repeat‐containing 2 (BIRC2), is a member of the IAP family, which consists of three motifs: Caspase Recruitment Domain (CARD), Really Interesting New Gene (RING), and Baculovirus IAP Repeat (BIR).^12^ In general, BIRC2 participates in the programmed cell death pathways, thereby regulating cell differentiation, cell survival, and signal transduction, possessing a wide spectrum of actions.^13^ Currently, the abnormal expression of BIRC2 has been discovered in multiple diseases, and BIRC2 is demonstrated to regulate the progression of various diseases, including cancers, infection, and cerebral ischemia.^14^, ^15^, ^16^ An integrated microarray analysis based on differentially expressed genes in RA identified BIRC2 as an upregulated RA‐specific gene. Meanwhile, BIRC2 acted as a hub gene of the RA‐specific protein–protein interaction network, suggesting that BIRC2 might play a critical role during RA development.^17^ However, the specific molecular mechanisms concerning the involvement of BIRC2 in RA have not been delineated.

Coincidently, it was found from the String website (https://cn.string-db.org/) that BIRC2 and tumor necrosis factor receptor 1‐associated death domain protein (TRADD) interacted with each other. TRADD is an adaptor molecule that binds to the TNF receptor (TNFR) to form complex I, followed by activating the nuclear factor (NF)‐κB and cell death signaling.^18^, ^19^ In particular, TRADD was revealed to be highly expressed in the peripheral blood mononuclear cells (PBMCs) of RA patients, highlighting the potential role of TRADD during RA development,^20^ whereas the specific regulatory mechanism of TRADD in RA is lacking.

Therefore, this study aims to explore the specific regulation of BIRC2/TRADD during the progression of RA, as well as their interaction, providing possible targets for the target treatment of patients with RA.

## 2 METHODS AND MATERIALS

### 2.1 Cell culture and induction

Human chondrocytes C28/I2 cells (#SCC043) were provided by Merck, and maintained in regular Dulbecco's modified Eagle's medium supplemented with 10% fetal bovine serum (FBS; Gibco) and 1% penicillin‐streptomycin (Gibco) at 37°C in a humidified incubator with 5% CO_2_. C28/I2 cells were stimulated by 10 μg/mL of lipopolysaccharide (LPS; Sigma‐Aldrich) to construct an in vitro RA cellular model as previously described.^21^

### 2.2 Quantitative real‐time polymerase chain reaction (qPCR)

Total RNA was isolated from cells employing Trizol reagent (Invitrogen). A total of 1 μg RNA was reverse‐transcribed into cDNA strictly in line with the guidelines of PrimeScript™ RT MasterMix kit (Invitrogen). Subsequently, qPCR was carried out utilizing the SYBR Green Mastermix kit (Takara). Gene expression was calculated by 2^−△△Ct^ method and normalized to β‐actin.

### 2.3 Western blot

Total protein was isolated from cells employing radioimmunoprecipitation assay (RIPA) reagent (Beyotime Biotechnology), followed by quantification using the Bradford assay kit (Bio‐Rad Laboratories). Total 30 μg of protein was subjected to 12% sodium dodecyl sulfate‐polyacrylamide gel electrophoresis gels, and the separated proteins were transferred onto polyvinylidene difluoride membranes. After being blocked with skimmed milk, the membranes were probed at 4°C overnight with primary antibodies, followed by incubation with the horseradish peroxidase‐conjugated secondary antibody at room temperature for 2 h. Eventually, the signals were developed with an enhanced chemiluminescence kit (Sigma‐Aldrich), and finally quantified using ImageJ software (NIH).

### 2.4 Cell transfection

For the BIRC2 knockdown, the small interfering RNA (siRNA) targeting BIRC2 (si‐BIRC2‐1 and si‐BIRC2‐2) were constructed by GenePharma. For the TRADD overexpression, the cDNA sequence of TRADD was cloned into the pcDNA3.1 vector (pcDNA‐TRADD). The empty vectors served as their negative controls (si‐NC and pcDNA‐NC), respectively. The above vectors were transfected into C28/I2 cells in line with the protocols of Lipofectamine 3000 reagent (Invitrogen). 48 h posttransfection, cells were harvested for subsequent experiments.

### 2.5 Cell counting kit‐8 (CCK‐8) assay

1 × 10^4^ C28/I2 cells were inoculated into 96‐well plates at 37°C with 5% CO_2_ and induced by LPS for 24 h. 10 μL of CCK‐8 solution (Dojingdo) was added to each well for a further incubation for 2 h. The absorption at 450 nm was measured under a microplate reader (Bio‐Rad Laboratories).

### 2.6 Flow cytometry analysis

Cell necroptosis was determined using flow cytometry analysis. In brief, C28/I2 cells were washed with pre‐cooled PBS and resuspended in a binding buffer. Subsequently, cells were stained with Annexin and PI for 15 min at room temperature away from the light. Eventually, cell necroptosis was determined using a FACSAria flow cytometry system (BD Bioscience).

### 2.7 Measurement of reactive oxygen species (ROS), malondialdehyde (MDA), superoxide dismutase (SOD), and glutathione peroxidase (GSH‐Px)

Cells were incubated with 5 μM of dichlorofluorescin‐diacetate (DCFH‐DA; Sigma‐Aldrich) reagent for 1 h. After washing, the intracellular ROS level was detected utilizing a spectrofluorophotometer (Shimadzu Corporation) at wavelengths of 488 nm excitation and 525 nm emission. In addition, the contents of MDA, SOD, and GSH‐Px in the cultured medium of each group were determined with the application of their corresponding commercial kits from Nanjing Jiancheng Bio Company by measuring the absorbance at 532, 450, and 412 nm, respectively.

### 2.8 Enzyme‐linked immunosorbent assay (ELISA)

The concentrations of inflammatory cytokines, including interleukin (IL)‐1β, IL‐6, and tumor necrosis factor (TNF)‐α, in the culture medium of different groups were determined using their corresponding ELISA kits from R&D Systems in accordance with the respective manuals.

### 2.9 Co‐immunoprecipitation (CoIP) assay

To investigate the interaction between BIRC2 and TRADD, total protein was isolated from cells employing RIPA reagent (Beyotime Biotechnology), followed by incubation with anti‐IgG antibody and anti‐BIRC2/anti‐TRADD antibody. The untreated proteins acted as input control. Subsequently, protein A/G PLUS‐Agarose beads (cat. no. sc‐2003; Santa Cruz Biotechnology) were added to generate an immune complex. The immunoprecipitated protein complex was boiled and denatured, followed by western blot assay as aforementioned to detect the precipitated protein, applying anti‐TRADD and anti‐BIRC2 antibodies, respectively.

### 2.10 Statistical analysis

All data were analyzed with Graphpad Prism software and were presented as mean ± standard deviation (SD) from at least three independent experiments. The distribution of variables was checked with Shapiro–Wilk tests. Student's *t*‐test was utilized to compare the difference between two groups, while one‐way analysis of variance assay followed by Tukey's post hoc test was utilized for more than two groups. A *p*‐value less than .05 was regarded as statistically significant.

## 3 RESULTS

### 3.1 BIRC2 was upregulated in LPS‐induced C28/I2 cells

**Figure 1:**
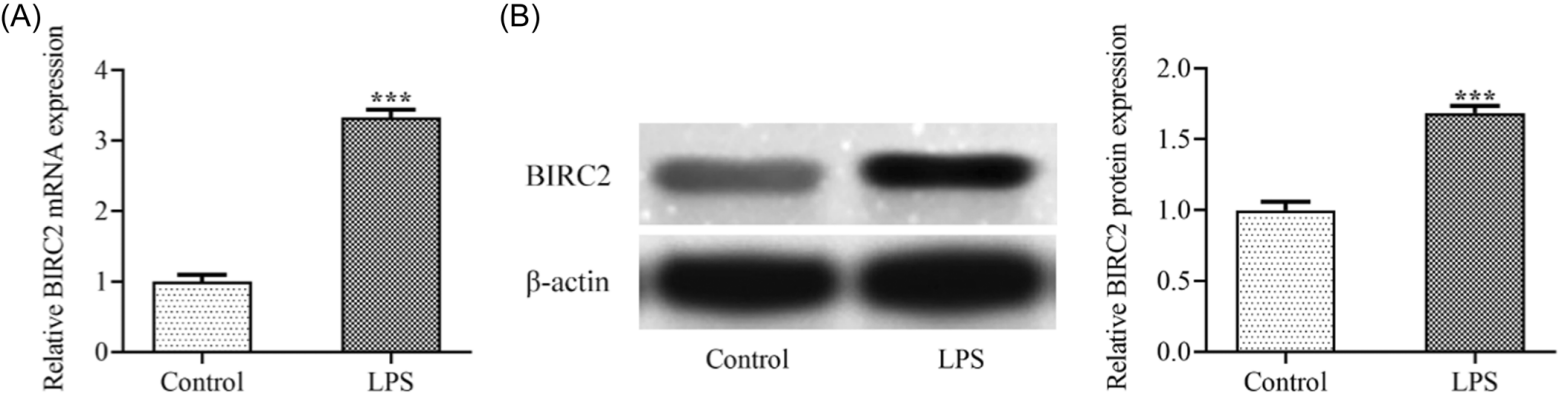
BIRC2 was upregulated in LPS‐induced C28/I2 cells. C28/I2 cells were stimulated by LPS to construct an in vitro RA cellular model. The (A) mRNA level and (B) protein expression of BIRC2 were examined using qPCR and western blot, respectively. ***p < .001. BIRC2, baculoviral IAP repeat‐containing 2; LPS, lipopolysaccharide; RA, rheumatoid arthritis.

First, C28/I2 cells were stimulated by LPS to establish an in vitro RA cellular model. It was observable from Figure 1A,B that compared with the Control, both mRNA level and protein expression of BIRC2 were significantly elevated after LPS induction, demonstrating that BIRC2 was highly expressed in in vitro RA cellular model.

### 3.2 BIRC2 knockdown elevated cell viability while suppressed necroptosis in LPS‐induced C28/I2 cells

**Figure 2:**
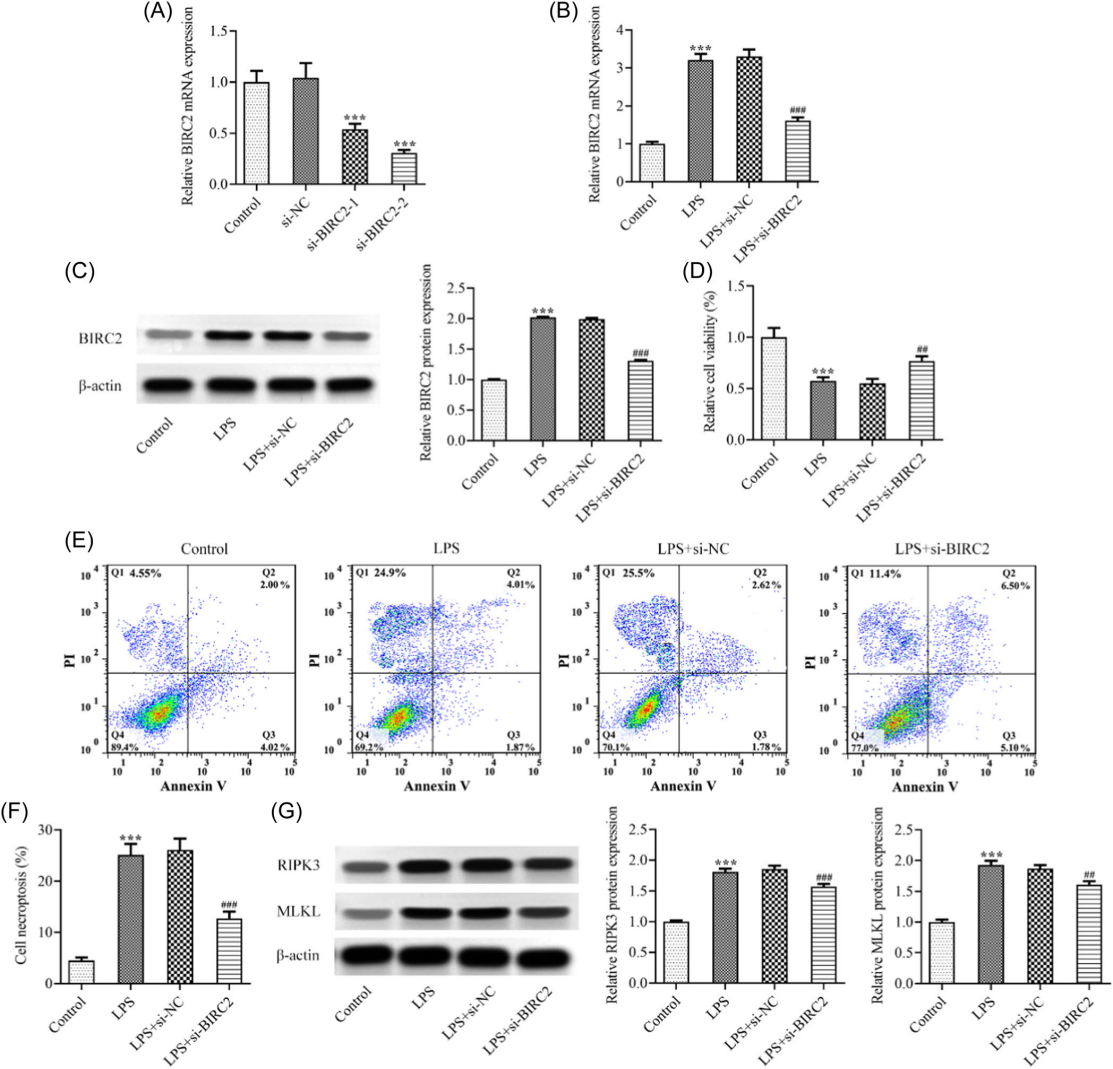
BIRC2 knockdown elevated cell viability while suppressing necroptosis in LPS‐induced C28/I2 cells. (A) C28/I2 cells were transfected with si‐NC or si‐BIRC2‐1/2, and the mRNA level of BIRC2 was examined using qPCR. ***p < .001 vs. si‐NC. The transfected or un‐transfected C28/I2 cells were stimulated by LPS, and the (B) mRNA level and (C) protein expression of BIRC2 were examined using qPCR and western blot, respectively. (D) CCK‐8 assay was performed to detect cell viability. (E and F) Flow cytometry analysis was performed to examine cell necroptosis. (G) The protein expression of RIPK3 and MLKL was measured by western blot assay. ***p < .001 vs. Control; ^##^ *p* < .01, ^###^ *p* < .001 vs. LPS+si‐NC. BIRC2, baculoviral IAP repeat‐containing 2; CCK‐8, Cell Counting Kit‐8; LPS, lipopolysaccharide; mRNA, messenger RNA; qPCR, quantitative real‐time polymerase chain reaction.

To explore the regulatory role of BIRC2 in RA, cell transfection was conducted. As presented in Figure 2A, compared with the si‐NC group, the mRNA level of BIRC2 in si‐BIRC2‐1 and si‐BIRC2‐2 groups was greatly reduced, demonstrating a successful knockdown of BIRC2 in C28/I2 cells. Attributed to a relatively high transfection efficacy, si‐BIRC2‐2 was selected for the following experiments. Thereafter, the transfected or un‐transfected C28/I2 cells were stimulated by LPS. As expected, the upregulated BIRC2 expression caused by LPS was inhibited following BIRC2 knockdown (Figure 2B,C). Compared with the LPS+si‐NC group, BIRC2 knockdown exhibited a great improvement of cell viability in LPS‐induced C28/I2 cells (Figure 2D). The flow cytometry assay revealed that LPS caused a remarkably increase of necroptosis in C28/I2 cells, which was partly abolished by BIRC2 knockdown (Figure 2E,F). Meanwhile, necroptosis‐related proteins, including receptor‐interacting protein kinase 3 (RIPK3) and mixed lineage kinase domain‐like protein (MLKL), were measured by western blot assay. The results showed that the protein expression of RIPK3 and MLKL was hugely increased upon LPS induction, which was then partly repressed following BIRC2 knockdown (Figure 2G).

### 3.3 BIRC2 knockdown alleviated oxidative stress and inflammation in LPS‐induced C28/I2 cells

**Figure 3:**
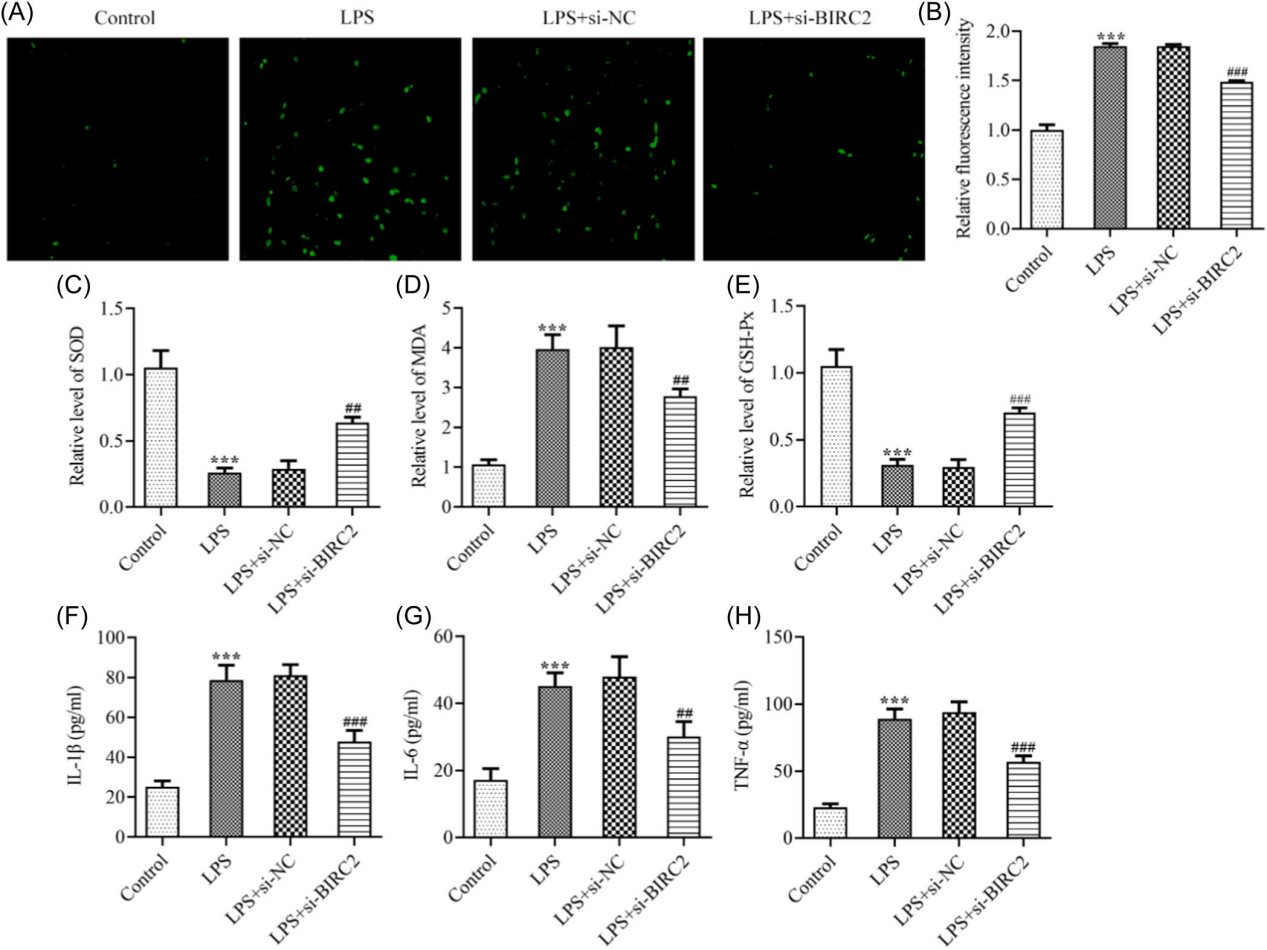
BIRC2 knockdown alleviated oxidative stress and inflammation in LPS‐induced C28/I2 cells. (A and B) The transfected or un‐transfected C28/I2 cells were stimulated by LPS, and the intracellular ROS level was detected utilizing DCFH‐DA reagent. The contents of (C) MDA, (D) SOD, and (E) GSH‐Px in cultured medium of each group were determined with the application of their corresponding commercial kits. The concentrations of (F) IL‐1β, (G) IL‐6, and (H) TNF‐α were measured their corresponding ELISA kits. ***p < .001 vs. Control; ^##^ *p* < .01, ^###^ *p* < .001 vs. LPS+si‐NC. BIRC2, baculoviral IAP repeat‐containing 2; DCFH‐DA, dichlorofluorescin‐diacetate; ELISA, enzyme‐linked immunosorbent assay; GSH‐Px, glutathione peroxidase; IL, interleukin; LPS, lipopolysaccharide; MDA, malondialdehyde; ROS, reactive oxygen species; SOD, superoxide dismutase.

In addition, the oxidative stress and inflammation following LPS stimulation in C28/I2 cells were also assessed. It was observable from Figure 3A,B that compared with the Control, LPS greatly triggered ROS activity in C28/I2 cells, while BIRC2 knockdown partly reversed this change. Meanwhile, LPS also greatly elevated MDA levels and reduced SOD and GSH‐Px levels in C28/I2 cells, demonstrating the occurrence of oxidative stress following LPS stimulation; however, these changes were greatly hindered by BIRC2 knockdown, evidenced by the reduced MDA level and restored SOD and GSH‐Px levels (Figure 3C–E). Moreover, the elevated pro‐inflammatory cytokines, including IL‐1β, IL‐6, and TNF‐α induced by LPS in C28/I2 cells were greatly reduced by BIRC2 knockdown (Figure 3F–H), suggesting that BIRC2 knockdown could attenuate LPS‐caused inflammation.

### 3.4 TRADD was upregulated in LPS‐induced C28/I2 cells and BIRC2 positively regulated TRADD expression

**Figure 4:**
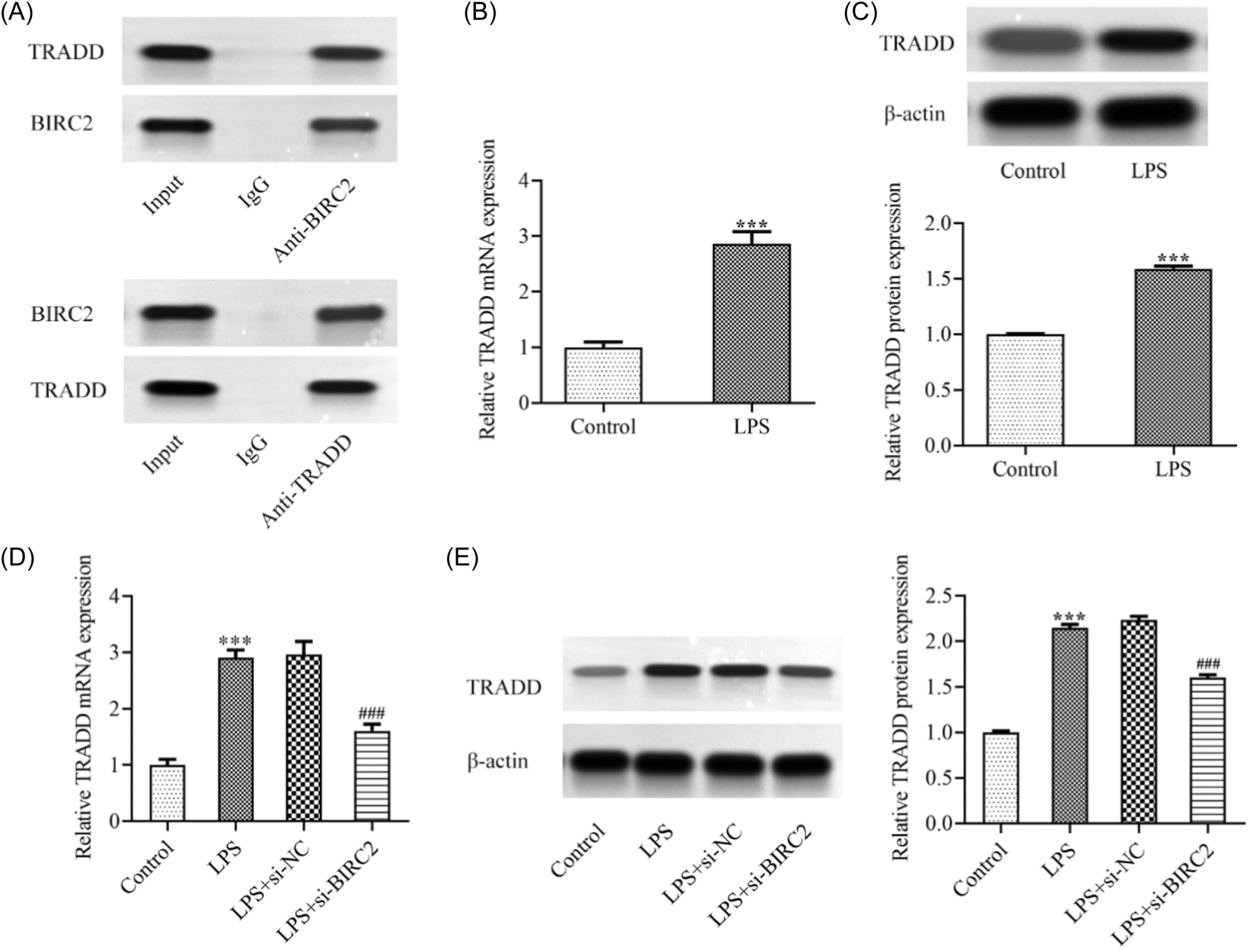
TRADD was upregulated in LPS‐induced C28/I2 cells and BIRC2 positively regulated TRADD expression. (A) The interaction between BIRC2 and TRADD was verified by CoIP assay. The (B) mRNA level and (C) protein expression of TRADD in LPS‐induced C28/I2 cells were examined using qPCR and western blot, respectively. ***p < .001. The si‐BIRC2‐transfected or un‐transfected C28/I2 cells were stimulated by LPS, and the (B) mRNA level and (C) protein expression of TRADD were examined using qPCR and western blot, respectively. ***p < .001 vs. Control; ^###^ *p* < .001 vs. LPS+si‐NC. BIRC2, baculoviral IAP repeat‐containing 2; CoIP, co‐immunoprecipitation; LPS, lipopolysaccharide; mRNA, messenger RNA; qPCR, quantitative real‐time polymerase chain reaction; TRADD, tumor necrosis factor receptor 1‐associated death domain protein.

To further explore the molecular mechanism of BIRC2 during the progression of RA, it was discovered that BIRC2 and TRADD interacted with each other, which was then verified by CoIP assay in Figure 4A. Subsequently, it was found from Figure 4B,C that no matter mRNA level or protein expression of TRADD was also greatly upregulated in LPS‐induced C28/I2 cells. In addition, the results in Figure 4D,E demonstrated that BIRC2 knockdown could downregulate TRADD expression in LPS‐induced C28/I2 cells.

### 3.5 TRADD overexpression abolished the attenuation of BIRC2 knockdown in LPS‐induced C28/I2 cells

**Figure 5:**
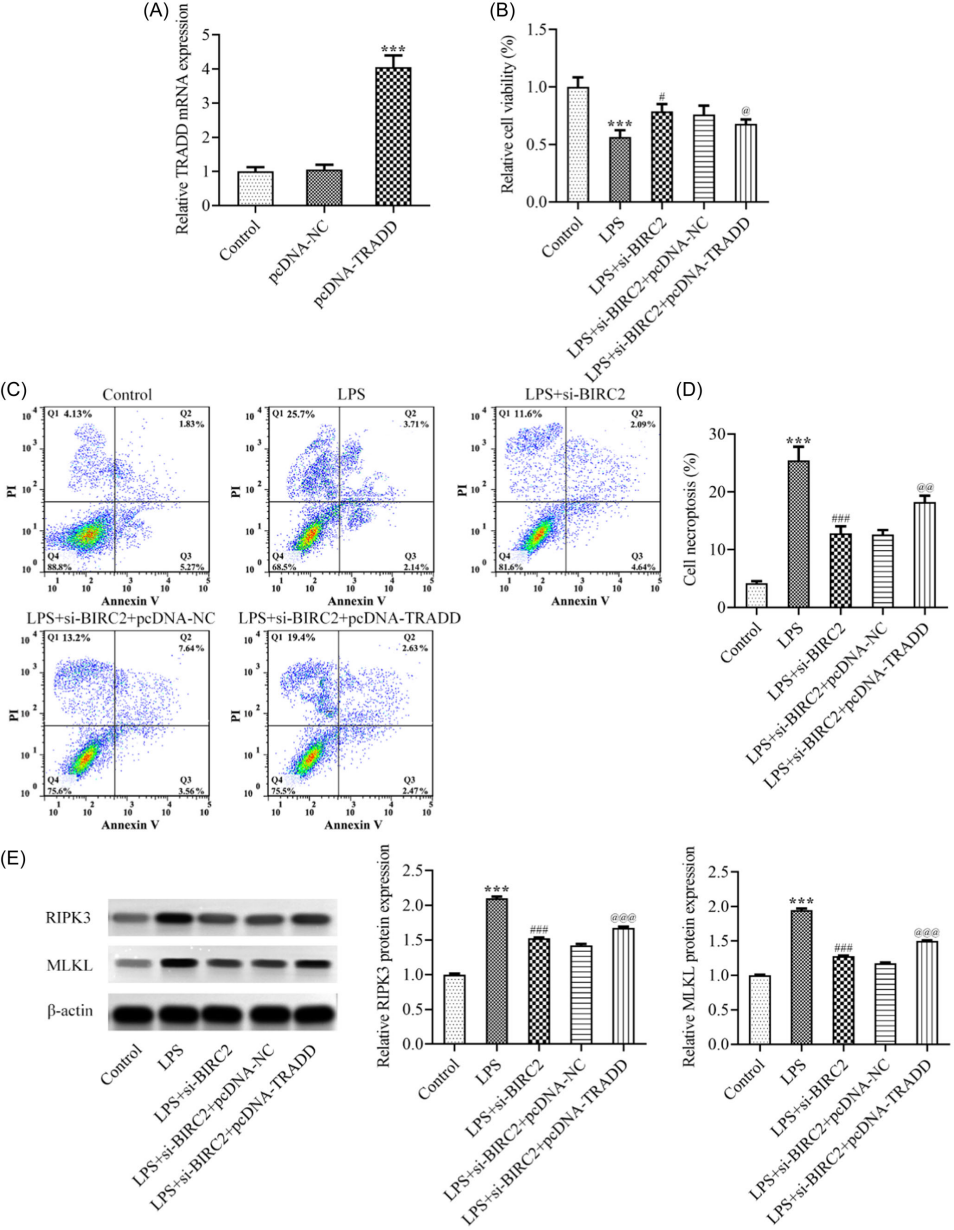
TRADD overexpression abolished the attenuation of BIRC2 knockdown on cell viability loss and necroptosis in LPS‐induced C28/I2 cells. (A) C28/I2 cells were transfected with pcDNA‐NC or pcDNA‐TRADD, and the mRNA level of TRADD was examined using qPCR. ***p < .001 vs. pcDNA‐NC. (B) C28/I2 cells were transfected with si‐BIRC2 alone or cotransfected with si‐BIRC2 and pcDNA‐NC/pcDNA‐TRADD, followed by LPS stimulation, and CCK‐8 assay was performed to detect cell viability. (C and D) Flow cytometry analysis was performed to examine cell necroptosis. (E) The protein expression of RIPK3 and MLKL was measured by western blot assay. ***p < .001 vs. Control; ^#^ *p* < .05, ^###^ *p* < .001 vs. LPS; p@ @ < .05, p@@ < .01, p@@@ < .001 vs. LPS+si‐BIRC2+pcDNA‐NC. BIRC2, baculoviral IAP repeat‐containing 2; CCK‐8, Cell Counting Kit‐8; LPS, lipopolysaccharide; mRNA, messenger RNA; qPCR, quantitative real‐time polymerase chain reaction; TRADD, tumor necrosis factor receptor 1‐associated death domain protein.

**Figure 6:**
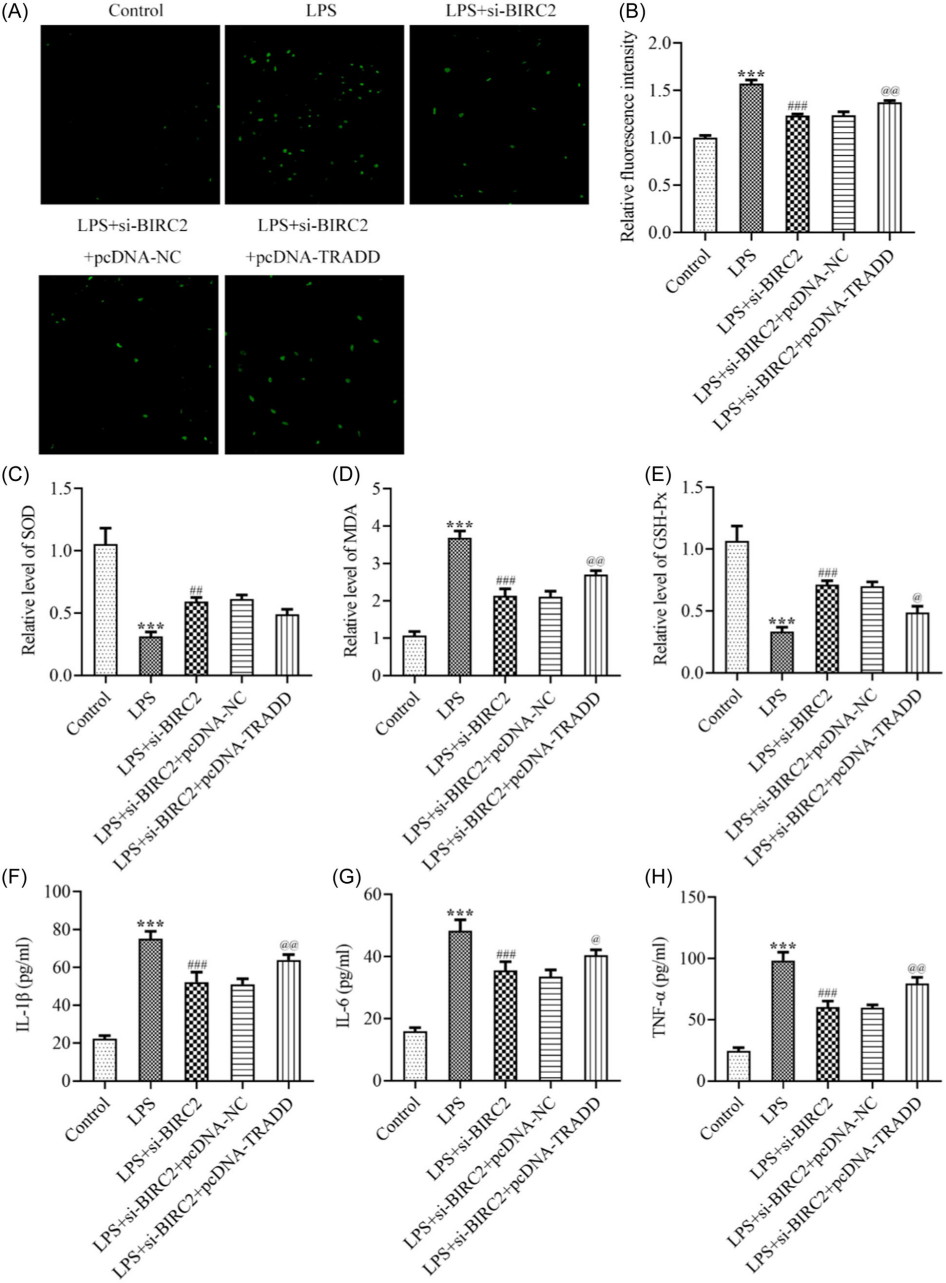
TRADD overexpression abolished the attenuation of BIRC2 knockdown on oxidative stress and inflammation in LPS‐induced C28/I2 cells. (A and B) C28/I2 cells were transfected with si‐BIRC2 alone or cotransfected with si‐BIRC2 and pcDNA‐NC/pcDNA‐TRADD, followed by LPS stimulation, and the intracellular ROS level was detected utilizing DCFH‐DA reagent. The contents of (C) MDA, (D) SOD, and (E) GSH‐Px in cultured medium of each group were determined with the application of their corresponding commercial kits. The concentrations of (F) IL‐1β, (G) IL‐6, and (H) TNF‐α were measured using their corresponding ELISA kits. ***p < .001 vs. Control; ^#^ *p* < .05, ^###^ *p* < .001 vs. LPS; p@ < .05, p@@ < .01, p@@@ < .001 vs. LPS+si‐BIRC2+pcDNA‐NC. BIRC2, baculoviral IAP repeat‐containing 2; DCFH‐DA, dichlorofluorescin‐diacetate; ELISA, enzyme‐linked immunosorbent assay; GSH‐Px, glutathione peroxidase; IL, interleukin; LPS, lipopolysaccharide; MDA, malondialdehyde; SOD, superoxide dismutase; TNF‐α, tumor necrosis factor‐α; TRADD, tumor necrosis factor receptor 1‐associated death domain protein.

Eventually, to clarify the regulation of BIRC2/TRADD during the progression of RA, C28/I2 cells were transfected with pcDNA‐TRADD to overexpress TRADD (Figure 5A). Then C28/I2 cells were transfected with si‐BIRC2 alone or cotransfected with si‐BIRC2 and pcDNA‐NC/pcDNA‐TRADD, followed by LPS stimulation. Subsequently, a series of cellular biological activities were assessed. The CCK‐8 assay revealed that the improved cell viability by BIRC2 knockdown was partly hindered by TRADD overexpression (Figure 5B). The results from flow cytometry analysis in Figure 5C,D revealed that the inhibitory effect of BIRC2 knockdown on cell necroptosis in LPS‐mediated C28/I2 cells was partly weakened by TRADD overexpression, which was further evidenced by the restored protein expression of RIPK3 and MLKL following additional TRADD overexpression (Figure 5E). In addition, the inhibitory effects of BIRC2 on the elevated ROS and MDA and the decreased SOD and GSH‐Px in LPS‐mediated C28/I2 cells were partly weakened by TRADD overexpression (Figure 6A–E), suggesting that TRADD overexpression could weaken the protective role of BIRC2 knockdown against oxidative stress during RA. In addition, TRADD overexpression also weakened the protective role of BIRC2 knockdown against inflammation, which was evidenced by the restored concentrations of IL‐1β, IL‐6, and TNF‐α following additional TRADD overexpression (Figure 6F–H).

## 4 DISCUSSION

RA is a chronic inflammation mediated by autoimmune response which affects approximately 1% of populations worldwide and seriously threatens the quality of people's physical and mental life.^22^ Thus, a better understanding of the pathogenesis of RA and developing effective targets for therapeutic strategies are urgently required.

In general, RA is mainly featured as joint lesions and degeneration of articular cartilage is the major contributor of joint dysfunction in patients with RA.^23^ LPS, a component of the outer envelope of gram‐negative bacteria, has been widely recognized as a pro‐inflammatory molecule that triggers the initiation of inflammation‐related diseases, including RA. Thus, an in vitro RA cell model was constructed using human chondrocytes C28/I2 exposed to LPS.^21^ In the present study, LPS greatly damaged chondrocytes, evidenced by the triggered inflammatory response and oxidative stress in C28/I2 cells. Meanwhile, LPS induced an upregulated expression of BIRC2, and LPS‐caused chondrocytes injuries were partly alleviated by BIRC2 knockdown, consistent with previously reported that both mRNA level and protein expression of BIRC2 was enhanced by LPS stimulation in human umbilical vein endothelial cells (HUVECs), while BIRC2 knockdown greatly inhibited LPS‐caused HUVEC autophagy and apoptosis,^24^ suggesting that BIRC2 knockdown exerted a protective role against LPS‐mediated cell injuries. Taken together, BIRC2 knockdown alleviated LPS‐caused inflammatory response and oxidative stress in chondrocytes, providing a potential target for RA treatment.

To further explore the molecular mechanism of BIRC2 during the progression of RA, we discovered that BIRC2 and TRADD interacted with each other, which was then verified by the CoIP assay in this study. TRADD is a unique adaptor molecular due to its key role in multiple downstream signaling events, including cell differentiation, survival, and death. In detail, the C‐terminal domain of TRADD can specifically interact with the intracellular death domain of TNFR1 to form a complex, creating a platform to activate NF‐κB and MAPK signaling.^18^, ^25^, ^26^ Meanwhile, NF‐κB and MAPK signaling is closely correlated to the inflammatory response of RA pathogenesis, and new drugs and therapies based on inhibiting NF‐κB and MAPK signaling have been demonstrated to exert a great anti‐inflammatory activity to prevent RA.^27^, ^28^, ^29^ In addition, BIRC2 acts as an E3 ubiquitin‐protein ligase which is a critical modulator of TNFR‐mediated signaling pathways, including NF‐κB.^30^, ^31^ Thus, BIRC2 may participate in TRADD‐mediated various cellular events. In this study, the protective role of BIRC2 knockdown against LPS‐induced inflammation, oxidative stress, and necroptosis in C28/I2 cells was partly diminished by TRADD overexpression, highlighting the critical role of BIRC2/TRADD axis in regulating RA progression.

Necroptosis, also named programmed necrosis, is a tightly adjusted inflammatory necrotizing cell death signaling pathway.^32^ MLKL is a kinase‐containing phosphorylation domain that is inactive under normal conditions. Upon pathological condition, necroptosis is triggered by the activation of death receptor, such as TNFR1. The activated TNFR1 mediates the activation of RIPK1 and RIPK3, promoting the phosphorylation of MLKL, which subsequently migrates towards the plasma membrane, causing changes in osmotic pressure and destroying the integrity of membrane structure, and eventually leading to the rupture of the plasma membrane.^33^, ^34^, ^35^ Thus, the triggered RIPK3‐MLKL is the critical downstream of the necroptotic pathway. Accumulating documents have recognized necroptosis as a crucial downstream target of the inflammatory process during RA progression, suggesting that necroptosis acts as an important regulator for RA.^34^, ^36^ Meanwhile, RIPK3 and MLKL were discovered to be highly expressed in RA patients and have been suggested to be promising therapeutic targets for the treatment of RA.^37^, ^38^ As expected, a triggered necroptosis and high levels of RIPK3 and MLKL proteins were discovered in LPS‐mediated C28/I2 cells in this study, while these elevations were greatly inhibited by BIRC2 knockdown, suggesting that BIRC2 knockdown could repress LPS‐caused necroptosis in C28/I2 cells. In addition, TRADD has been demonstrated to mediate RIPK1‐dependent necroptosis by regulating the RIPK3‐MLKL pathway, highlighting the critical role of TRADD in regulating necroptosis,^39^ which was also reflected in the present study that TRADD overexpression greatly weakened the inhibitory effect of BIRC2 knockdown on necroptosis in LPS‐mediated C28/I2 cells.

## 5 CONCLUSION

In summary, BIRC2 knockdown alleviated necroptosis, oxidative stress, and inflammation in LPS‐mediated C28/I2 cells, which might be attributed to the interaction between BIRC2 and TRADD, and BIRC2 knockdown might exert its functions through downregulating TRADD. Hence, this study provides potential therapeutic targets for the future treatment of RA.

## AUTHOR CONTRIBUTIONS

**Yanting Rao**: Data curation; methodology; project administration; software; validation; visualization; writing—original draft. **Shengjing Xu**: Data curation; methodology; software; validation; visualization. **Ting Lu**: Data curation; software; validation. **Yuanyuan Wang**: Data curation; methodology. **Manman Liu**: Data curation; methodology. **Wei Zhang**: Conceptualization; project administration; resources; supervision; writing—review & editing.

## CONFLICT OF INTEREST STATEMENT

The authors declare no conflict of interest.

## Data Availability

All data generated have been included in this article.
