# Correction to “Downregulation of BIRC2 Hinders the Progression of Rheumatoid Arthritis Through Regulating TRADD]”

**DOI:** 10.1002/iid3.70261

**Published:** 2025-09-11

Y. Rao, S. Xu, T. Lu, Y. Wang, M. Liu and W. Zhang, “Downregulation of BIRC2 hinders the progression of rheumatoid arthritis through regulating TRADD”, *Immunity, Inflammation and Disease*, no. 11 (2023): e978, https://doi.org/10.1002/iid3.978.

In paragraph 4 of the Materials and Methods section, the sequence information of si‐BIRC‐1 and si‐BIRC2 is missing. This should be modified as follows: For the BIRC2 knockdown, the small interfering RNA (siRNA) targeting BIRC2 (si‐BIRC2‐1 (5′‐CAGGGCTTTAAGTTAGTATTA‐3′) and si‐BIRC2‐2 (5′‐TAGGCGTTTCTGATAACACTA‐3′)) were constructed by GenePharma (Shanghai, China).

The authors apologize for this omission.

